# Impact of omega-3 fatty acids supplementation on the gene expression of peroxisome proliferator activated receptors-*γ*, *α* and fibroblast growth factor-21 serum levels in patients with various presentation of metabolic conditions: a GRADE assessed systematic review and dose–response meta-analysis of clinical trials

**DOI:** 10.3389/fnut.2023.1202688

**Published:** 2023-11-15

**Authors:** Amirhossein Ramezani Ahmadi, Fatemeh Shirani, Behnazi Abiri, Mansoor Siavash, Sasan Haghighi, Mojtaba Akbari

**Affiliations:** ^1^Isfahan Endocrine and Metabolism Research Center, Isfahan University of Medical Sciences, Isfahan, Iran; ^2^Obesity Research Center, Research Institute for Endocrine Sciences, Shahid Beheshti University of Medical Sciences, Tehran, Iran

**Keywords:** n-3 fatty acids, polyunsaturated fatty acids, PUFA, PPAR, fibroblast growth regulatory factor

## Abstract

There is some debate about the effects of omega-3 fatty acids on the regulation of adipose tissue related genes. This systematic review and meta-analysis aimed to evaluate the effects of omega-3 fatty acids supplementation on the gene expression of peroxisome proliferator activated receptors (*PPAR-α* and *PPAR-γ*) and serum fibroblast growth factor-21 (FGF-21) levels in adults with different presentation of metabolic conditions. To identify eligible studies, a systematic search was conducted in the Cochrane Library of clinical trials, Medline, Scopus, ISI Web of Science, and Google Scholar up to April 2022. Eligibility criteria included a clinical trial design, omega-3 fatty acids supplementation in adults, and reporting of at least one of the study outcomes. Effect sizes were synthesized using either fixed or random methods based on the level of heterogeneity. Fifteen studies met the inclusion criteria. Omega-3 fatty acids supplementation significantly increased the *PPAR-γ* (10 studies) and *PPAR-α* (2 studies) gene expression compared to the control group (WMD: 0.24; 95% CI: 0.12, 0.35; *p* < 0.001 and 0.09; 95% CI: 0.04, 0.13; p < 0.001, respectively). Serum FGF-21 (8 studies) levels exhibited no significant change following omega-3 fatty acids supplementation (*p* = 0.542). However, a dose–response relationship emerged between the dose of omega-3 fatty acids and both *PPAR-γ* gene expression and serum FGF-21 levels. Overall, this study suggests that omega-3 fatty acids supplementation may have positive effects on the regulation of adipose tissue related genes in patients with various presentation of metabolic condition. Further research is needed to validate these findings and ascertain the effectiveness of this supplementation approach in this population.

**Systematic Review Registration:**

https://www.crd.york.ac.uk/prospero/display_record.php?, CRD42022338344.

## Introduction

Omega-3 fatty acids have demonstrated a wide range of health benefits, including the capacity to reduce hypertriglyceridemia, adverse cardiovascular events, and the regulation of blood pressure, glucose tolerance, and nervous system functions. Moreover, omega-3 fatty acids have been associated with decreased insulin secretion, enhanced insulin resistance, and improved endothelial function. They also exhibit anti-inflammatory, antioxidant, and anti-thrombotic properties (1, 2). However, the precise mechanisms through which omega-3 fatty acids exert their metabolic effects remain incompletely understood. Previous studies have suggested that the metabolic effects of n-3 polyunsaturated fatty acids (PUFAs) involve the modulation of gene expression in adipose tissue (3). For example, omega-3 fatty acids are recognized as natural modulators of peroxisome proliferator activated receptors (*PPAR-α*, *PPAR-γ*, and *PPAR-δ*) and improvement of fibroblast growth factor-21 (FGF-21), but the whole mechanisms are not clear (4–8).

*PPAR-γ* and *PPAR-α* serve as nuclear receptors with pivotal roles in the regulating of lipid and glucose metabolism. PPAR-γ regulates adipocyte differentiation, lipid storage, and adipokine secretion. Defects in *PPAR-γ* function contribute to insulin resistance and obesity. Additionally, *PPAR-α* and FGF-21 promote fat oxidation and thermogenesis in adipose tissue, potentially counteracting metabolic abnormalities linked to obesity. The connection between *PPAR-γ*, *PPAR-α*, and serum levels of FGF-21 in the context of metabolic disorders such as type 2 diabetes (T2DM), non-alcoholic fatty liver disease (NAFLD), obesity, poly-cystic ovary syndrome (PCOS), gestational diabetes mellitus (GDM), cardiac disease, and dyslipidemia arises from their roles in regulating glucose and lipid metabolism, insulin sensitivity, inflammation, and oxidative stress. Modulating these pathways through PPARs activation or increased FGF-21 levels may hold therapeutic potential promise for these conditions.

Experimental studies showed that both fish oil and flaxseed oil up-regulate the expression of *PPAR*-α and *PPAR*-γ (9, 10). Rahmani et al. (11) observed a significant improvement in *PPAR*-γ gene expression following 12 weeks of fish oil supplementation in subjects with PCOS. Other studies investigated the impact of omega-3 fatty acids supplementation on the regulation of plasma FGF-21 levels and its role in modulating critical metabolic pathways in white adipose tissue.

FGF-21 is a novel metabolic regulator that is primarily produced by the liver (7, 12–14). Recently, it was described that omega-3 fatty acids can reduce circulating FGF-21 levels and enhance FGF-21 sensitivity, potentially through a *PPAR*-γ-dependent mechanism (15). Nevertheless, the outcomes of previous studies present conflicting findings (14, 16, 17). Consequently, the present study aimed to systematic review and meta-analysis clinical trials that evaluated the effect of omega-3 fatty acids supplementation on *PPAR-γ* and *PPAR-α* gene expression and serum FGF-21 levels in patients with various presentation of metabolic conditions. The results of this study could enhance our understanding of the metabolic actions of omega-3 fatty acids and offer insights into their potential therapeutic applications.

## Materials and methods

This research followed the PRISMA statement for systematic reviews and meta-analyzes. The systematic review protocol was registered in PROSPERO under the code CRD42022338344. Ethical approval for the study methodology was obtained from the ethics committee of Isfahan University of Medical Sciences (IR.ARI.MUI.REC.1400.135).

### Search strategy

Two researchers (BA and FS) independently conducted searches in various databases, including the Cochrane Library of clinical trials (CENTRAL), Medline, Scopus, ISI Web of Science, and Google Scholar for studies that investigated the effects of omega-3 fatty acids on the gene expression of *PPAR-γ, α* and serum levels of FGF-21 in individuals with different presentation of metabolic conditions. The search included all original papers published until April 2022. Various combinations of keywords and medical subject heading (MeSH) terms were used, including n-3 fatty acids, fish oil, n-3 oil, n-3 Polyunsaturated Fatty Acid, n-3 PUFA, alpha-Linolenic Acid, Docosahexaenoic Acids, Eicosapentaenoic Acid, DHA, EPA, ALA, omega 3, omega-3 fatty acids, peroxisome proliferator activated receptor, PPAR, Thiazolidinedione Receptor, NR1C3, FGF, fibroblast growth factor. There were no restrictions on publication year or language. Moreover, the reference lists of included studies were reviewed to identify any additional relevant studies. Two reviewers independently screened the titles and abstracts of the search results to select potentially relevant studies.

### Study selection

Full texts of studies aligned with the objectives of the present study were examined, and those meeting the eligibility criteria were included. The inclusion criteria consisted of studies written in English or Persian, studies involving omega-3 fatty acids supplementation, studies evaluating the gene expression of *PPAR-γ, α* or serum levels of the FGF-21 as study outcomes, and clinical trial study designs. Due to limited number of clinical trials available, we were unable to include a specific population group in our study. However, all human studies conducted in patients with metabolic conditions related to obesity, insulin sensitivity, and dyslipidemia were considered. The following reports were excluded: non-full-text articles, ecological studies, animal studies, observational studies, opinion articles, conference abstracts, review papers, editorials, studies not assessing relevant outcomes or populations, and studies using omega-3 fatty acids supplements in combination with other bioactive agents.

### Data extraction

The data extraction was independently conducted by two researchers (AR and MA). In the case of discrepancies, consensus was reached through cross-examination by MS. The extracted study characteristics included the first author’s name, year of publication, country, baseline age, body mass index (BMI), sample size, composition of the supplement and placebo, dose of omega-3 fatty acids, study duration, and study population. Additionally, mean ± SD values of serum FGF-21 and fold change of *PPAR-γ* and *PPAR-α* gene expression were derived from eligible studies at both the baseline and the end of the study.

### Assessment of risk of bias

Two independent researchers assessed the quality of the trials using the revised Cochrane risk of bias tool for randomized trials (RoB 2). The RoB2 evaluates various aspects of trial design, conduct, and reporting. The quality of the studies was categorized as “Low risk,” “High risk,” or expressed as having “Some concerns.”

### Statistical analysis

The mean difference ± SD was used as the effect size and was pooled using fixed method meta-analysis (inverse variance). In the presence of significant heterogeneity, the random method (Dersimonian-Laird) was employed to pool effect sizes. Heterogeneity between studies was evaluated using the *I*^2^ index and Cochrane’s Q test. *I*^2^ Interpretation is as follows: low if *I*^2^ is <30%, moderate if *I*^2^ is 30–75%, and high if *I*^2^ is >75% (18).

Subgroup analyzes were conducted based on age (≤55 or > 55 years), source of omega-3 fatty acids (fish oil or plant-based oil), and different population groups (diabetic or non-diabetic/ obese or overweight/dyslipidemia or non-dyslipidemia) to explore potential variations in study results. Sensitivity analysis and meta-regression were performed to further investigate the effects of different variables on study outcomes. One-stage non-linear dose–response meta-analyzes were conducted using the DRMETA module developed by Nicola Orsini (19) to examine the effect of omega-3 fatty acids supplementation on the gene expression of *PPAR-γ* and serum FGF-21 levels. Publication bias was assessed using Begg’s rank correlation, Egger’s linear regression, and visual inspection of the funnel plot. If publication bias was detected, the Trim and Fill method was applied to adjust for intervention effects. All analyzes were performed using Stata, version 17 (Stata Crop, College Station, TX, United States) and a *p*-value of <0.05 was considered statistically significant.

## Results

### Characteristics of the studies

**Figure 1:**
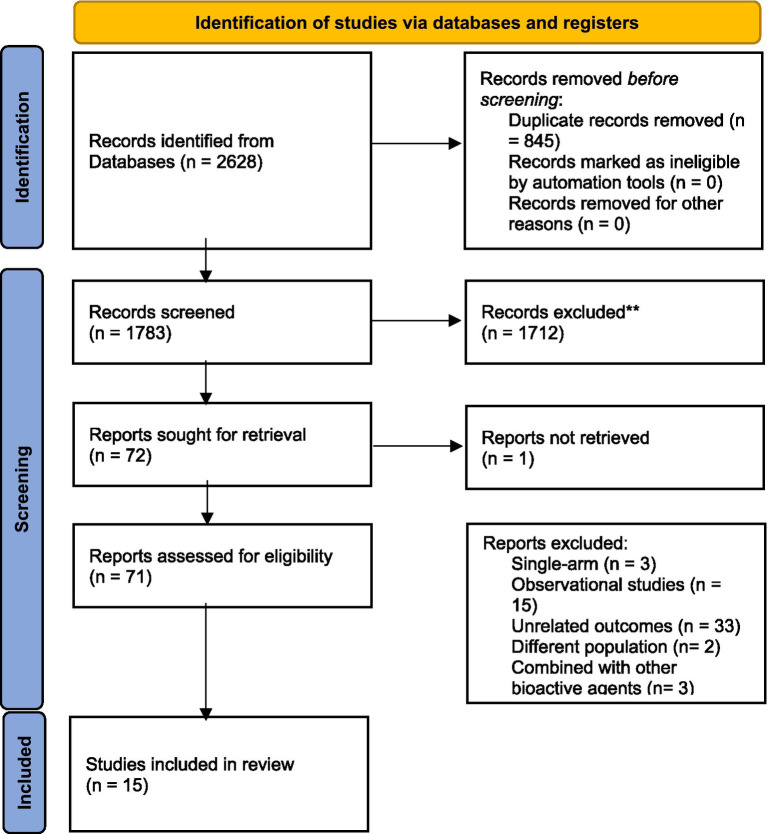
PRISMA Flow diagram of the study selection process.

**Figure 2:**
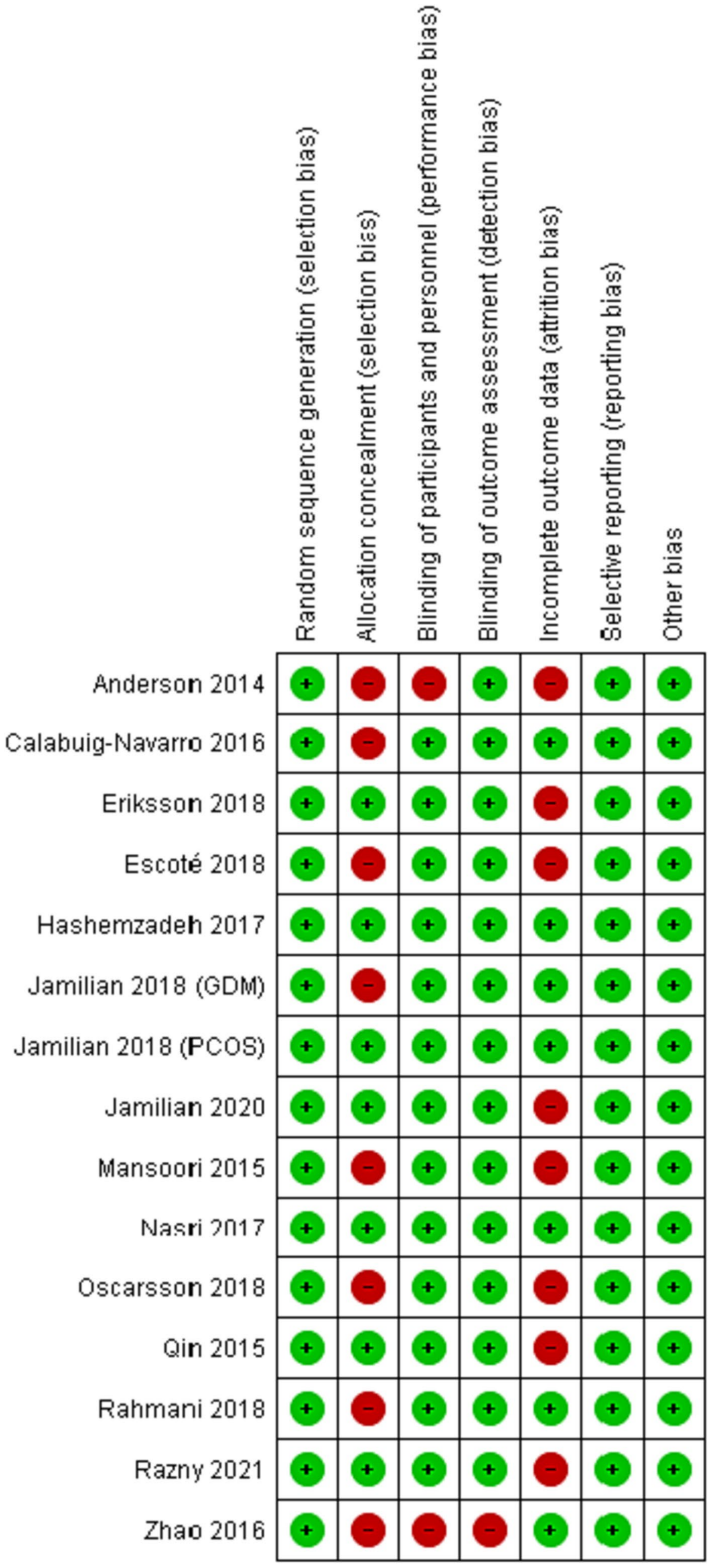
Risk of bias summary of included studies.

The initial electronic search resulted in 1783 studies after removing duplicates (see Figure 1). Following the title and abstract screening, 1712 studies, including review articles, study protocols, animal studies, or studies not related to the purpose of the present study, were excluded. A total of 71 studies underwent eligibility assessment, and 55 studies were excluded for various reasons, such as being single-arm studies, observational studies, studies not evaluating the gene expression of *PPAR-γ, α* or serum levels of FGF-21, studies not conducted in patients with metabolic diseases or insulin impairment (obesity, diabetes, dyslipidemia, polycystic ovary syndrome, and heart disease), and studies using a combination of omega-3 fatty acids with other nutrients or bioactive agents. Studies that reported outcomes other than gene expression (such as the activity of *PPAR-γ*) were also excluded. Ultimately, 15 studies met the inclusion criteria and provided sufficient data for meta-analysis (2, 5, 7, 8, 11–14, 16, 20–25). The general characteristics of the included studies are summarized in Table 1. These studies were conducted in various locations, including the United States (8, 21), Iran (5, 11, 20, 22–25), China (2, 12), Sweden (13, 14), Spain (7), and Poland (16). The intervention durations ranged from 3 to 24 weeks. Ten studies utilized fish oil as the source of omega-3 fatty acids (7, 8, 11–14, 16, 20, 21, 24), while five studies used plant-sources (flaxseed or perilla oil) (2, 5, 22, 23, 25). *PPAR*-γ,*α* gene expression were primarily assessed using peripheral blood mononuclear cells (PBMCs), with two studies using atrial myocardium and placental tissue samples (8, 21). The risk of bias assessment is summarized in Figure 2, with the most common issues related to allocation concealment (selection bias) and incomplete outcome data (attrition bias).

**Table 1:** Characteristics of included studies.

First author, year	Country	Participants, *n* (% male)	Study duration, weeks	Age, years	BMI, kg/m^2^	Omega-3 FA dose, mg/d	Omega-3 FA type	Placebo type	Population group
Anderson, 2014 (1)	United States	24 (66)	3	64.4	31.1	3,400	Fish oil	No treatment	Patients before elective cardiac surgery
Mansoori, 2015 (2)	Iran	68 (NR)	8	55.8	28.3	2,400	DHA-rich fish oil	Paraffin	T2DM patients
Qin, 2015 (3)	China	70 (73)	12	45.1	26.2	4,000	Fish oil	Corn oil	Patients with NAFLD characteristics associated with hyperlipidemia
Calabuig-Navarro, 2016 (4)	United States	33 (0)	24	27.3	33.4	2,000	Fish oil	Wheat germ oil	Obese and overweight woman
Zhao, 2016 (5)	China	26 (58)	24	48.9	32.5	8,000	Perilla oil	No treatment	Obese patients
Hashemzadeh, 2017 (6)	Iran	60 (NR)	12	59.6	30.4	1,000	Flaxseed oil	Paraffin	T2DM Patients with CHD
Nasri, 2017 (7)	Iran	60 (0)	12	26.8	27.1	2,000	Flaxseed oil	Paraffin	PCOS
Eriksson, 2018 (8)	Sweden	75 (71)	12	65.4	31.2	4,000	OM-3CA	NR	T2DM patients
Rahmani, 2018 (13)	Iran	40 (0)	12	26.6	26.4	2000	Fish oil	NR	PCOS
Jamilian, 2018 (11)	Iran	40 (0)	12	23.3	27.6	1,000	Flaxseed oil	Paraffin	PCOS
Jamilian, 2018 (10)	Iran	40 (0)	6	30.6	27.5	2,000	Fish oil	NR	GDM
Escoté, 2018 (9)	Spain	57 (0)	10	38.4	32.2	1,341	Fish oil	Sunflower oil	Obese and overweight woman
Oscarsson, 2018 (12)	Sweden	51 (59)	12	59.7	29.8	4,000	OM-3CA	NR	Overweight or obese individuals with NAFLD and hypertriglyceridemia
Jamilian, 2020 (14)	Iran	51 (0)	6	29.0	28.1	2,000	Flaxseed oil	Sunflower oil	GDM
Razny, 2021 (15)	Poland	64 (47)	12	41.3	32.9	1,800	Fish oil	Corn oil	Overweight or obesity (with abdominal obesity)

NR, not reported; BMI, body mass index; FA, fatty acid; T2DM, type 2 diabetes mellitus; NAFLD, non-alcoholic fatty liver disease; CHD, coronary heart disease; PCOS, polycystic ovary syndrome; GDM, gestational diabetes mellitus.

### The effect of omega-3 fatty acids supplement on *PPAR-γ* gene expression

**Figure 3:**
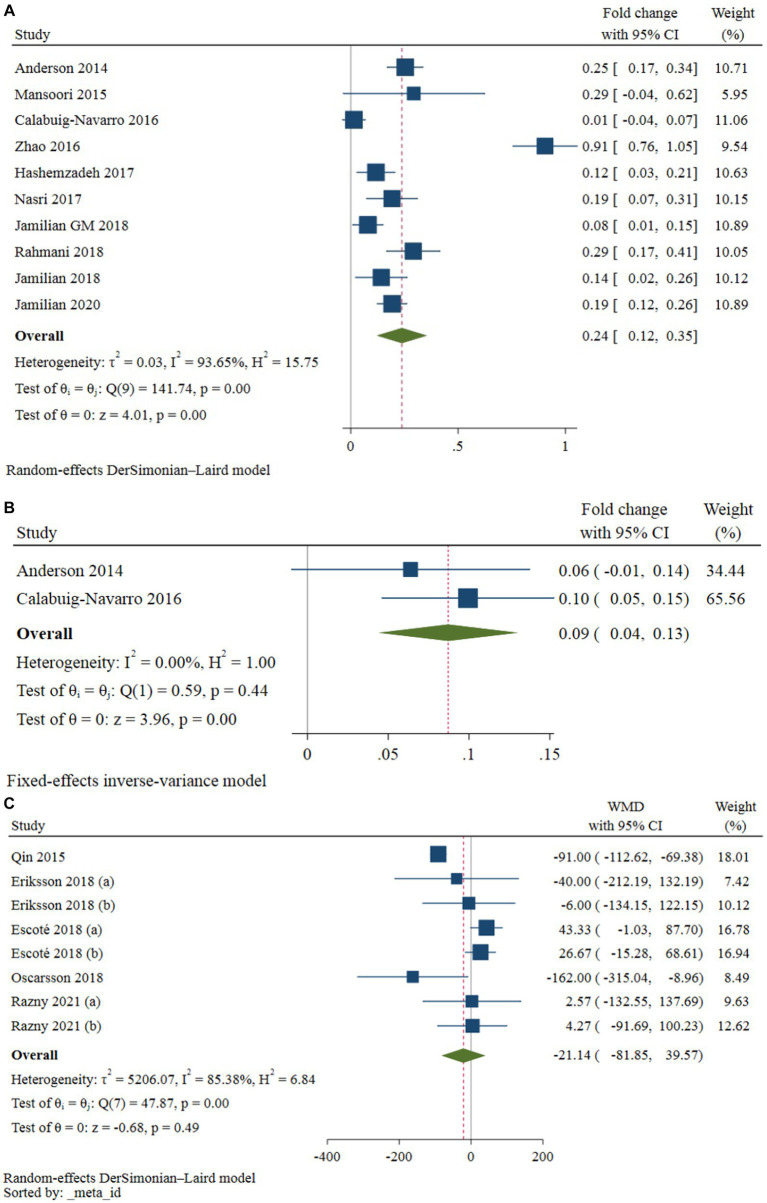
Forest plot of studies evaluating the effect of omega-3 fatty acids supplementation on the expression of *PPAR-γ* **(A)**, *PPAR-α* **(B)**, and serum levels of FGF-21 **(C)** in patients with metabolic risk factors.

**Figure 4:**
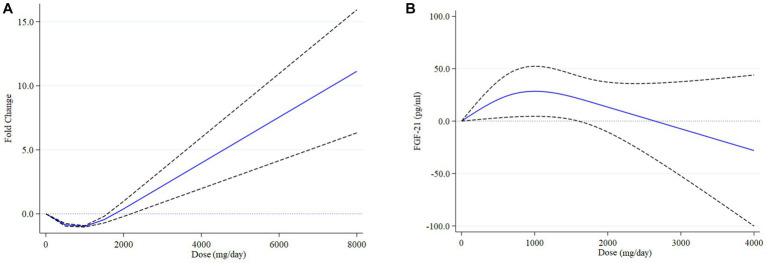
Non-linear dose–response relationship between omega-3 fatty acids supplement (mg/d) and difference of fold change of *PPAR-γ* expression **(A)** and mean difference of serum FGF-21 levels **(B)** in patients with metabolic risk factors.

Ten studies, comprising 224 intervention and 218 control participants, evaluated the effect of omega-3 fatty acids supplementation on gene expression of *PPAR-γ* (2, 5, 8, 11, 20–25). The results of the meta-analysis (see Table 2) indicated that omega-3 fatty acids supplementation significantly increased *PPAR-γ* gene expression compared to the control group (difference in fold change: 0.24; 95% CI: 0.12, 0.35; *p* < 0.001; see Figure 3A). However, a high level of heterogeneity was observed among the studies (*I*^2^ = 93.65; *p* < 0.001). A non-linear dose–response relationship was identified between the dose of omega-3 fatty acids and *PPAR-γ* gene expression (*p* < 0.001; see Figure 4A). Subgroup analysis revealed moderate, non-significant heterogeneity in populations with an average age over 55 years (*I*^2^ = 62.17; *p* = 0.071) and in diabetic patients (*I*^2^ = 51.77; *p* = 0.101). However, the between-subgroups heterogeneity test was not significant for age, presence of diabetes, weight status, and source of omega-3 fatty acids supplementation (*p* > 0.05). Sensitivity analysis did not lead to changes in the results when excluding one study at a time. The Galbraith plot (see Supplementary Figure S1) indicated five studies as potential sources of heterogeneity (2, 8, 11, 21, 24). Meta-regression analysis revealed a direct association between dose (*p* = 0.002) and the percentage of male participants in the study (*p* = 0.033) with changes in *PPAR-γ* gene expression following omega-3 fatty acids supplementation.

**Table 2:** Overall estimates of meta-analysis for the effect of omega-3 fatty acids supplement on expression of *PPAR-γ,α* and serum FGF-21 levels in patients with metabolic risk factors.

Outcome	Subgroups	Studies, *n*	Reference	WMD (95% CI)	*p*	I^2^ (%)	P heterogeneity	P heterogeneity between subgroups
*PPAR-γ, fold change*		10		0.24 (0.12, 0.35)	<0.001	93.65	<0.001	–
Age	≤55 years	7	(2, 5, 11, 21, 23–25)	0.25 (0.10, 041)	0.002	95.53	<0.001	0.587
	>55 years	3	(8, 20, 22)	0.20 (0.08, 0.31)	0.001	62.17	0.071	
Presence of diabetes	Yes	4	(20, 22, 24, 25)	0.14 (0.07, 0.20)	<0.001	51.77	0.101	0.154
	No	6	(2, 5, 8, 11, 21, 23)	0.29 (0.09, 0.50)	0.005	96.28	<0.001	
Weight status	Overweight	6	(5, 11, 20, 23–25)	0.18 (0.11, 0.24)	<0.001	56.35	0.043	0.339
	Obese	4	(2, 8, 21, 22)	0.31 (0.03, 0.59)	0.026	97.69	<0.001	
Source of omega-3	Fish oil	5	(8, 11, 20, 21, 24)	0.16 (0.04, 0.29)	0.007	88.32	<0.001	0.269
	Plant-based oil	5	(2, 5, 22, 23, 25)	0.30 (0.09, 0.52)	0.005	95.48	<0.001	
*PPAR-α, fold change*		2		0.09 (0.04, 0.13)	<0.001	0.0	0.442	–
*Serum FGF-21, pg/ml*		8	(7, 12–14, 16)	-21.13 (−81.84, 39.56)	0.494	85.38	<0.001	–
Age	≤55 years	5	(7, 12, 16)	−4.93 (−78.89, 69.03)	0.896	91.17	<0.001	0.333
	>55 years	3	(13, 14)	−64.41 (−159.28, 30.46)	0.183	17.90	0.296	
Presence of diabetes	Yes	2	(13)	−18.12 (−120.92, 84.68)	0.730	0.0	0.756	0.955
	No	6	(7, 12, 14, 16)	−21.69 (−91.45, 48.08)	0.542	89.46	<0.001	
Presence of dyslipidemia	Yes	2	(12, 14)	−92.38 (−113.79, −70.98)	<0.001	0.0	0.368	<0.001
	No	6	(7, 13, 16)	27.02 (−0.35, 54.4)	0.053	0.0	0.889	
Weight status	Overweight	2	(12, 14)	−92.38 (−113.79, −70.98)	<0.001	0.0	0.368	<0.001
	Obese	6	(7, 13, 16)	27.02 (−0.35, 54.4)	0.053	0.0	0.889	

FGF, fibroblast growth factor-21; PPAR, peroxisome proliferator activated receptors; WMD, weighted mean difference.

### The effect of omega-3 fatty acids supplement on *PPAR-α* gene expression

Only two studies investigated the effect of omega-3 fatty acids supplementation on *PPAR-α* gene expression. It was demonstrated that omega-3 fatty acids supplementation significantly increased the *PPAR-α* gene expression compared to the control group (difference in fold change: 0.09; 95% CI: 0.04, 0.13; *p* < 0.001; see Figure 3B). There was no obvious heterogeneity between these studies (I^2^ = 0.0, *p* = 0.442). Due to the limited number of studies, subgroup, sensitivity, meta-regression, and dose–response analyzes were not possible (Table 3).

**Table 3:** Meta-regression for the effect of baseline characteristics on the association between omega-3 fatty acids supplementation and expression of *PPAR-γ* and serum FGF-21 levels in patients with metabolic risk factors.

Variable	*N*	Coefficient	SE	*p*-value	*I*^2^ (%)	P heterogeneity
*PPAR-γ*						
Age	10	0.005	0.004	0.228	93.53	<0.001
Study duration	10	0.012	0.009	0.201	94.21	<0.001
Male percent	10	0.004	0.002	0.033	92.56	<0.001
BMI	10	0.025	0.028	0.370	94.21	<0.001
Dose	10	0.357	0.114	0.002	90.65	<0.001
DHA/EPA ratio	5	0.053	0.073	0.471	90.31	<0.001
FGF-21						
Age	8	−3.12	2.74	0.256	80.38	<0.001
Study duration	8	−50.52	14.92	0.001	26.65	0.225
Male percent	8	−1.70	0.26	<0.001	0.00	0.526
BMI	8	18.80	2.90	<0.001	0.00	0.456
Dose	8	−0.05	0.006	<0.001	0.00	0.788
DHA/EPA ratio	8	3.98	16.18	0.806	87.31	<0.001

BMI, body mass index; DHA, docosahexaenoic acid; EPA, eicosapentaenoic acid; FGF, fibroblast growth factor-21; PPAR, peroxisome proliferator activated receptors; SE, standard error.

### The effect of omega-3 fatty acids supplement on serum FGF-21 levels

Five studies, with a total of 160 participants in the intervention group and 157 participants in the placebo group, provided eight effect sizes for evaluating the impact of omega-3 fatty acids supplementation on serum FGF-21 levels. The meta-analysis revealed no-significant difference in the change in serum FGF-21 between the omega-3 fatty acids and control groups (WMD: -21.13; 95% CI: −91.45, 48.08; *p* = 0.542; Figure 3C). However, a dose–response relationship was observed between the dose of omega-3 fatty acids and serum FGF-21 levels (*p* = 0.042; Figure 4B), with the highest FGF-21 level observed at a dose of 1,000 mg/day (WMD: 28.48; 95% CI:4.58, 52.37). A high level of heterogeneity was observed between studies (*I*^2^ = 85.38, *p* < 0.001). The Galbraith plot (Supplementary Figure S2) identified the studies of Qin et al. (12) and Escoté et al. (7) as sources of heterogeneity. A significant reduction following omega-3 fatty acids supplementation was observed in patients with dyslipidemia and overweight (WMD: −92.38; 95% CI: −113.79, −70.98; *p* < 0.001). Heterogeneity was not significant in the older age (P 0.296) and diabetic patient (P 0.756) subgroups. Subgroup analysis identified the presence of dyslipidemia and weight status as sources of heterogeneity (*p* < 0.001). The meta-regression analysis suggested that study duration, sex, BMI, and dose as sources of heterogeneity. A direct association was found between BMI and the mean difference in serum FGF-21. Additionally, an inverse association was observed between the mean difference in serum FGF-21 and study duration (*p* = 0.001), the percentage of male participants in the study (*p* < 0.001), and dose (*p* < 0.001). Sensitivity analysis did not provide any further information.

### Publication bias

Visual inspection of the funnel plot, Begg’s non-parametric rank correlation test (*p* = 0.07 and 0.386, respectively), and the regression-based Egger test (*p* = 0.06 and 0.659) did not reveal significant publication bias in the studies evaluating the effect of omega-3 fatty acids supplementation on *PPAR-γ* gene expression and serum FGF-21 levels (Supplementary Figures S3, S4).

### The GRADE assessment

Table 4 provides the GRADE assessment profile of the study outcome. The evidence regarding the effect of omega-3 fatty acids supplementation on *PPAR-γ* gene expression was of “moderate” quality. The certainty of evidence was rated as “low” and “very low” for serum FGF-21 and *PPAR-α* gene expression, respectively.

**Table 4:** GRADE profile of omega-3 fatty acids supplementation on expression of *PPAR-γ,α* and serum levels of FGF-21 in patients with metabolic risk factors.

Certainty assessment							No of patients		Certainty	Importance
No of studies	Study design	Risk of bias	Inconsistency	Indirectness	Imprecision	Other considerations	Omega-3	control		
*PPAR-γ*										
10	Randomized trials	Not serious	Very serious^1^	Serious^2^	Not serious	Strong association dose response gradient	224	218	⨁⨁⨁◯ Moderate	Critical
*PPAR-α*										
2	Randomized trials	Serious^3^	Not serious	Serious^4^	Serious^5^	None	29	28	⨁◯◯◯ Very low	Important
FGF-21										
8	Randomized trials	Not serious	Very serious^1^	Not serious	Serious^6^	Dose response gradient	160	157	⨁⨁◯◯ Low	Important

CI, confidence interval; FGF, fibroblast growth factor-21; MD, mean difference; PPAR, peroxisome proliferator activated receptors.

1. Downgraded because I-squared was > 75%; 2. More than 50% of the population are female; 3. Downgraded because 50–70% of the studies had a high risk of bias; 4. Studies are from different population groups; 5. There is a small number of studies; 6. There is wide 95% confidence interval.

## Discussion

The primary objective of this research was to consolidate findings from existing clinical trials and assess the impact of omega-3 fatty acids supplementation on the expression of *PPAR-γ* and *PPAR-α* genes, and serum FGF-21 levels in patients with various presentation of metabolic conditions. The study included 15 clinical trials involving individuals with diverse health profiles, employing different doses and sources of omega-3 fatty acids as interventions, sometimes in conjunction with placebos as controls. The quality of evidence regarding the effect of omega-3 fatty acids supplementation on *PPAR-γ* gene expression was of “moderate” quality. However the level of certainty of evidence was “low” for serum FGF-21 and “very low” for *PPAR-α* gene expression, respectively.

The meta-analysis results indicated a significant elevation in *PPAR-γ* gene expression due to omega-3 fatty acids supplementation when compared to the control group. Two studies that explored the effect the impact of omega-3 supplementation on *PPAR-α* gene expression also reported significant increases compared to the control group. However, the meta-analysis did not reveal a significant difference in the change of serum FGF-21 between the groups receiving omega-3 fatty acids and control. A non-linear dose–response relationship was observed between the dose of omega-3 and serum FGF-21 levels, with the highest levels observed at a dose of 1,000 mg/day and declining in higher doses. Subgroup analysis showed a significant reduction in patients with dyslipidemia and overweight following omega-3 supplementation.

Previous experimental studies have demonstrated that omega-3 PUFAs activate members of the PPAR superfamily (26, 27), and increase *PPAR-α* mRNA expression in subcutaneous adipose tissues in obese adolescents after 12 weeks of of omega-3 fatty acids supplementation (28). *PPAR-γ*, a member of the nuclear receptor superfamily, plays a pivotal role in regulating glucose and lipid metabolism, immune function, and inflammation (11). It also influences adipocyte function, differentiation, insulin and lipid metabolism, and lipid storage (22). Down-regulation of *PPAR-γ* is involved in the pathological process of various diseases, including diabetes, atherosclerosis and cancer (11). The regulatory effect of *PPAR-γ* activity extends to genes like carboxykinase, glucose-6-phosphatase, and the fatty acid transporter-1, ultimately decreasing free fatty acids production and enhancing insulin sensitivity (29).

Previous research has indicated that that the intake of 1,000 mg of omega-3 fatty acids from flaxseed oil twice daily for 12 weeks can increase *PPAR-γ* gene expression in women with PCOS (23). Similarly, in a study (25) aimed at evaluating the impact of omega-3 fatty acids from flaxseed oil on genetic and metabolic profiles in women with GDM, a significant enhancement in *PPAR-γ* was observed. Moreover, supplementing GDM women with 1,000 mg/day of fish oil for 6 weeks was found to enhance *PPAR-γ* gene expression (24). Linseed oil was also shown to elevate *PPAR-γ* gene expression in goats (30). However, a study involving T2DM patients demonstrated that *PPAR-γ* gene expression did not respond to fish oil supplementation of 2,400 mg/day after 8 weeks (20). It appears that omega-3 fatty acids may affect metabolic conditions through pathways beyond PPAR regulation, including modulating cyclin-dependent kinase inhibitor 2A and telomerase activity (31). Additionally, no significant change in *PPAR-γ* gene expression was observed in the bovine uterus after exposure to EPA (32). The variations in findings among studies may be attributed to differences in baseline characteristics of the study subjects, the varying dosages of fish oil supplements, and the study duration. The mechanisms and regulation of *PPAR-γ* signaling by fish oil remain largely unknown. It appears that omega-3 fatty acids are the natural ligands of *PPAR-γ*, and they are able to activate the production of *PPAR-γ* (11).

FGF-21 levels are typically reduced in patients with T2DM treated with anti-diabetes medications (33). However, animal studies have indicated that elevated FGF-21 levels or treatment with FGF-21 leads to improved glucose and lipid metabolism, weight loss, and NAFLD (34, 35). On the other hand, *in vivo* omega-3 PUFAs supplementation (mixture of EPA and DHA) induce the expression and release of FGF-21 (36). In mice, dietary omega-3 fatty acids prevent the increase in plasma FGF-21 levels induced by a high-fat diet (37). However, it was observed that EPA may prevent FGF-21 from declining during weight loss (7). In contrast, fish oil was found to reduce FGF-21 levels in patients with NAFLD, suggesting that fish oil may influence the amelioration of FGF-21 resistance (12). There is some evidence indicating that the elevated FGF-21 levels may not be the primary mechanism through which omega-3 PUFAs alleviate metabolic disorders. Omega-3 PUFAs and EPA alone have been reported to induce thermogenic activation, which in turn increases FGF-21 levels according to some investigations (7). Therefore, the impacts of omega-3 PUFAs on FGF-21 remain unclear and may depend on the specific tissue or metabolic status. Further studies are warranted to substantiate the beneficial impact of fish oil on the FGF-21 resistance in patients with impaired glucose metabolism, and to evaluate the underlying mechanisms for FGF-21 as a therapeutic target.

Strengths of this study include being the first systematic review and meta-analysis on the impact of omega-3 fatty acids supplementation on *PPAR-γ, α*, and serum FGF-21 levels in patients with metabolic conditions. The absence of publication bias and inclusion of only clinical trials are also strengths. However, potential limitations of the study include the inherent variations among the original trials, such as different health conditions (such as NAFLD, T2DM, PCOS, GDM, overweight/obese with or without dyslipidemia, abdominal obesity), varying BMI ranges, divergent doses of omega-3 fatty acids, differing intervention durations, other concurrent interventions, and the relatively limited number of studies included in subgroup analyzes. Moreover, the omega-3 form animal and plant-based source were pooled together despite the structural difference. Therefore, it is imperative to interpret the results with caution and acknowledge that mentioned factors could have contributed to the observed heterogeneity. This heterogeneity could have affected the validity and generalizability of the findings. Although we attempted to evaluate the impact of these factors on our overall findings in subgroup analyzes, we acknowledge it is not possible to account for this heterogeneity directly in our analyzes. Overall, the presence of significant heterogeneity among studies is an important limitation that should be acknowledged and considered when interpreting the results. Additionally, most of the studies in dose–response meta-analysis had only two arms. Future research should consider using more biologically relevant exposure levels, such as absorbed DHA/EPA levels, and examining how intervention type may affect the result. Also, it should be aimed to minimize heterogeneity by employing consistent methodologies, standardizing the dose and source of omega-3 fatty acids, controlling for confounding variables, and ensuring a more homogeneous participant selection process.

## Conclusion

Overall, omega-3 fatty acids supplementation in patients with various presentation of metabolic conditions significantly improved gene expression of *PPAR-γ, α*, but it did not affect serum FGF-21 levels. However, there was a dose–response relationship between the dose of omega-3 fatty acids and serum FGF-21 levels, with the highest level observed at a dose of 1,000 mg/day. Furthermore, a significant reduction was observed in patients with dyslipidemia and overweight following omega-3 fatty acids supplementation. This meta-analysis provides valuable insight into the therapeutic implications of omega-3 fatty acids in disorders related to metabolic conditions, but further research is needed to determine its effectiveness and safety on every specific disease, separately.

## Data availability statement

The original contributions presented in the study are included in the article/Supplementary material, further inquiries can be directed to the corresponding author.

## Author contributions

AA, MS, and MA: study conception and design. AA, BA, and SH: data collection. MA, FS, and BA: analysis and interpretation of results. AA, FS, SH, and MS: draft manuscript preparation. All authors reviewed the results and approved the final version of the manuscript.
