# Supplementary material for: Impact of omega-3 fatty acids supplementation on the gene expression of peroxisome proliferator activated receptors-γ, α and fibroblast growth factor-21 serum levels in patients with various presentation of metabolic conditions: a GRADE assessed systematic review and dose–response meta-analysis of clinical trials
Source: Front Nutr. 2023 Nov 15;10:1202688. doi: 10.3389/fnut.2023.1202688 (PMC10684744; doi:10.3389/fnut.2023.1202688)
Supplement: Supplementary file 1 [file Data_Sheet_1.docx]

**Supplementary files**


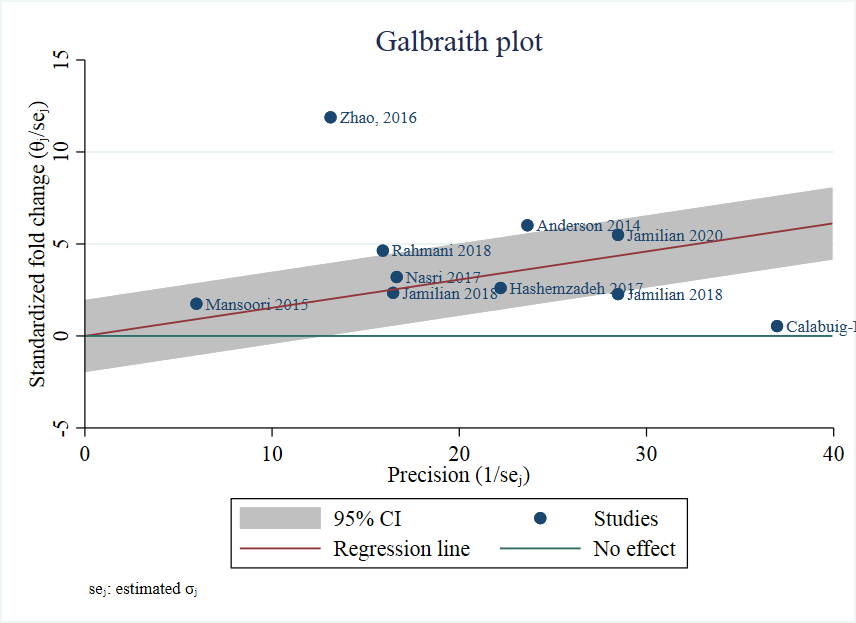


Figure S1. Galbraith plot of the studies evaluating the effect of omega-3 fatty acid supplementation on *PPAR-γ* gene expression.


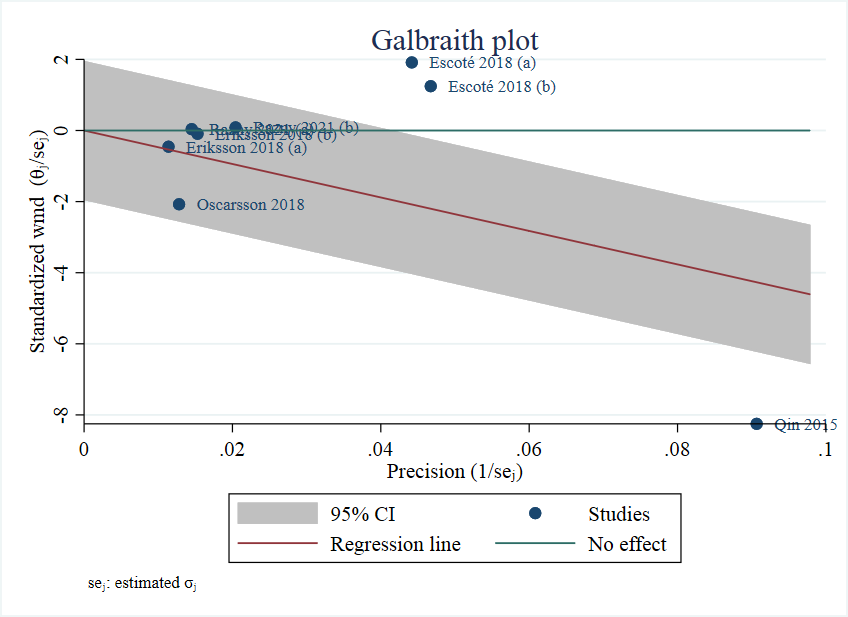


Figure S2. Galbraith plot of the studies evaluating the effect of omega-3 fatty acid supplementation on serum FGF-21 levels.


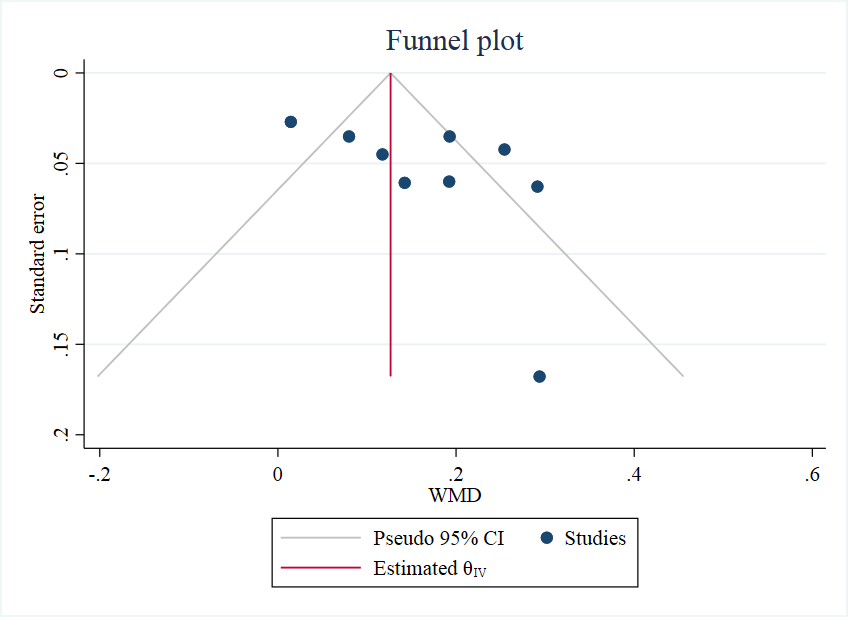


Figure S3. Funnel plot to assess publication bias of the studies evaluating the effect of omega-3 fatty acid supplementation on *PPAR-γ* gene expression.


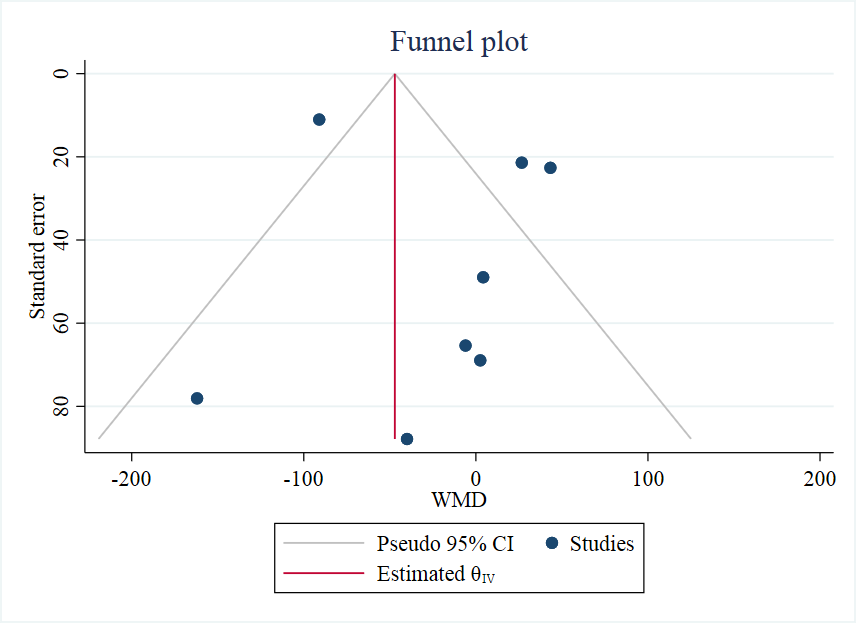


Figure S4. Funnel plot to assess publication bias of the studies evaluating the effect of omega-3 fatty acid supplementation on serum FGF-21 levels.
